# Do older individuals who are diagnosed with cancer have worse physical performance prior to diagnosis compared to matched controls? A longitudinal cohort study

**DOI:** 10.1186/s12877-018-0850-z

**Published:** 2018-07-18

**Authors:** S. M. L. M. Looijaard, M. S. Slee-Valentijn, L. N. Groeneveldt, D. J. H. Deeg, M. Huisman, A. B. Maier

**Affiliations:** 1 Section of Gerontology and Geriatrics, Department of Internal Medicine, VU University Medical Center, De Boelelaan 1117, 1081 HV Amsterdam, The Netherlands; 2 Center of Excellence in Geriatric Rehabilitation, Cordaan, Box 1103, 1000 BC Amsterdam, the Netherlands; 3 Department of Epidemiology & Biostatistics, EMGO+ Institute for Health and Care Research, VU University Medical Center, Van der Boechorststraat 7, 1081 BT Amsterdam, The Netherlands; 4 Department of Sociology, Vrije Universiteit Amsterdam, Van der Boechorstraat 7, 1081 BT Amsterdam, The Netherlands; 5 Department of Human Movement Sciences, @AgeAmsterdam, Amsterdam Movement Sciences, Vrije Universiteit Amsterdam, Van der Boechorststraat 7, 1081 BT Amsterdam, The Netherlands; 6 Department of Medicine and Aged Care, @AgeMelbourne, The Royal Melbourne Hospital, University of Melbourne, City & Royal Park Campus, 34-54 Poplar Road, Parkville, Melbourne, Victoria 3052 Australia

**Keywords:** Aged, Older, Geriatrics, Oncology, Cancer, Neoplasms, Physical performance

## Abstract

**Background:**

Impaired physical performance is highly prevalent in older cancer patients and is associated with cancer-related outcomes such as mortality and chemotherapy-related toxicity. Physical performance might already decline prior to the cancer diagnosis due to undiagnosed disease. This study aimed to assess whether the physical performance of community-dwelling individuals prior to cancer diagnosis is worse compared to matched controls who are not diagnosed with cancer.

**Methods:**

The study sample was selected from the Longitudinal Aging Study Amsterdam, a longitudinal study on a nationally representative sample of the Dutch older population. Physical performance of initially cancer-free individuals aged 55–84 years who were diagnosed with cancer during 10 or 20 years of follow-up was compared to the physical performance of controls who were not diagnosed with cancer. For controls, the physical performance measurements of the cycle with a median age closest to the cancer group were used. The time interval between physical performance measurements and the report of cancer was 2 to 4 years. Groups were compared using logistic and linear regression analysis.

**Results:**

The study sample included 1735 individuals with a median age of 68.7 [interquartile range 63.3–76.4] years. During follow-up, 414 (23.9%) individuals were diagnosed with cancer. Handgrip strength, gait speed, chair stand ability, chair stand test time and ability to put on and take off a cardigan did not differ between groups. Individuals prior to cancer diagnosis were more likely to complete the tandem balance test.

**Conclusions:**

Physical performance of individuals 2 to 4 years prior to report of cancer diagnosis is not lower compared to controls. This suggests that physical performance may not be influenced by cancer before diagnosis.

## Background

Decreased physical performance is highly prevalent in older patients who have been diagnosed with cancer [1–6]. Almost half of older cancer patients experience problems with walking [7, 8] while this is approximately 20–30% in the general older population [9, 10]. Moreover, approximately 25% of older cancer patients have mobility impairment measured by a prolonged timed up and go test [11–13] with a median time of 17–24 s [12, 13] while the mean time to perform the timed up and go test in the general older population is 8.7–10 s [14–16]. The importance of physical performance in older cancer patients has been highlighted by its predictive power for clinically relevant outcomes such as mortality and chemotherapy-related toxicity [13, 17–21].

Physical performance of individuals who have been diagnosed with cancer shows a greater decline than matched controls not suffering from cancer [5, 22], which may be caused by cachexia due to the disease [23] or chemotherapy treatment [12, 24, 25]. The majority of individuals with cancer who did not receive chemotherapy treatment in the previous 4 weeks, show a decrease in body weight [26, 27]. This weight loss might be a consequence of muscle wasting and therewith influencing physical performance. Self-reported physical ability to perform several daily routine activities 3 months prior to cancer diagnosis has been reported to be better than after initial treatment 8 weeks after diagnosis [28]. Another study showed that self-reported physical performance within 1 year after cancer diagnosis was lower compared to a group without cancer [22]. In the 3 years before cancer diagnosis the self-reported physical performance was only lower in the group with lung cancer compared to individuals without cancer [22]. These findings are based on subjective data and it is unclear whether physical performance was objectively lower or when decline took place. Self-reported physical performance can be influenced by a variety of (subjective) factors unrelated to actual physical performance such as state of mind, and are probably less sensitive to minor changes than objective physical performance measures. Evidence about the occurrence of objective decline in physical performance prior to cancer diagnosis is therefore important; it may be used as an indicator for undiagnosed cancers and it may be predictive of recovery relating to cancer treatment.

The aim of this study was to examine whether objectively measured physical performance of individuals prior to the diagnosis of cancer was worse compared to individuals who did not develop cancer in a large community sample of older adults. We hypothesized that physical performance of individuals prior to cancer diagnosis would be lower than the physical performance of individuals without cancer due to a decline in muscle mass and fatigue even before cancer diagnosis.

## Methods

### Study design and procedures

The study sample was selected from the Longitudinal Aging Study Amsterdam (LASA), a longitudinal study on a nationally representative sample of the Dutch older population among 3107 community dwelling individuals aged 55–84 years (first cohort, 1992–1993) and 1002 individuals aged 55–64 years (second cohort, 2002–2003) [29]. Individuals were followed about every 3 years since these baseline measurements. Thus after the baseline measurement of cohort one in 1992–1993, a second cohort was added in 2002-2003 and the latest follow-up included in this study took place in 2011-2012. Detailed information on data procedures and collection has been published elsewhere [29, 30]. The study has been approved by the Medical Ethics Committee of the VU University Medical Center in Amsterdam.

### Study sample

Individuals were divided into a cancer group and control group based on self-report of having cancer during any of the follow-up measurements. To determine the physical performance of cancer patients prior to their diagnosis, the measurement cycle before they reported to have cancer for the first time was used for analysis. After the baseline and follow-up interviews of 2001–2002, 2005–2006 and 2008–2009, information on presence of cancer was also obtained from general practitioners by use of a questionnaire. As self-reports of cancer were fairly accurate compared to reports of general practitioners (1992–1993 kappa = 0.64 (0.58–0.70) and 2008–2009 kappa = 0.64 (0.57–0.70), the cancer group in this study was based on self-report of cancer [31, 32]. Individuals were included in the cancer group if they reported cancer for the first time at any of the follow up measurements. Individuals were excluded if 1) they reported cancer at baseline (*n* = 372); 2) they did not participate in the most recent interview before reporting cancer (*n* = 9); 3) no physical performance tests were conducted during the interview before reporting cancer (*n* = 2). The number of individuals included in the cancer group was *N* = 414.

**Fig. 1:**
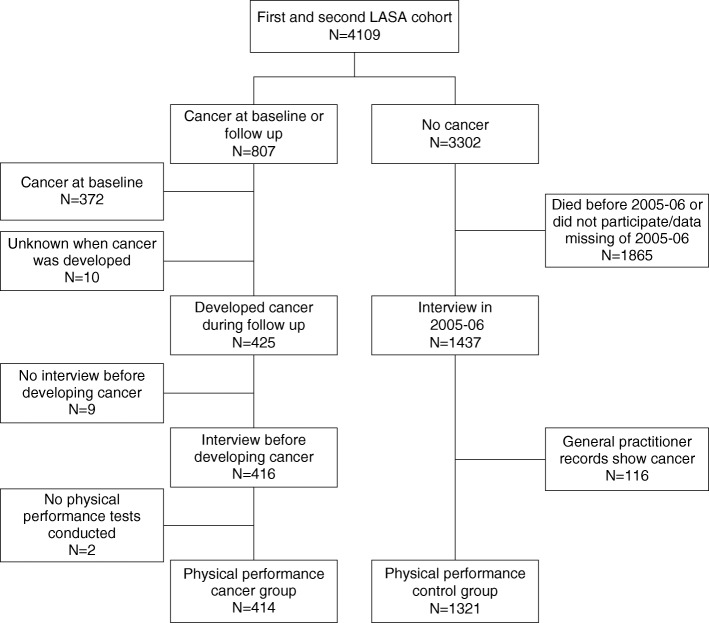
Flowchart of selection of study sample

The control group consisted of individuals who reported no cancer at baseline nor during the complete 10 (cohort 2) to 20 (cohort 1) years of follow-up. The measurement cycle of the year 2005–2006 was chosen as the physical performance measurement for the control group, since the median age of individuals in this cycle was nearest to the median age of the individuals diagnosed with cancer. Individuals were excluded from the control group if: 1) they died before the interview in 2005–2006, did not participate in the interview or data of the interview or physical performance measurements were missing in 2005–2006 (*n* = 1865); 2) they did not report presence of cancer at any of the interviews but the general practitioner did (*n* = 116), to maximize the probability that the controls were cancer free. The number of individuals included in the control group was *N* = 1321. Figure 1 illustrates the selection of the study sample from the cohort.

### Characteristics of study sample

Data on age, gender, lifestyle factors, anthropometry and health characteristics were included as covariates. Lifestyle variables included current smoking status and alcohol use. Current smoking status (never, former and current smoker) was dichotomized into yes (current smoker) or no (never or former smoker). Alcohol consumption was measured with an adaptation of the alcohol consumption index by Garretsen [33]. This index was categorized into (very) excessive alcohol use, light/moderate alcohol use and no alcohol use. Anthropometric measurements included height, weight and body mass index (BMI). Health characteristics included cognitive functioning, number of chronic diseases and number of medicines used per day. Cognitive functioning was measured by the Mini Mental State Examination (MMSE) [34]. Number of chronic diseases was obtained by asking explicitly about the presence of six prevalent somatic chronic diseases (chronic non-specific lung disease including asthma and chronic obstructive pulmonary disease, cardiac disease, peripheral arterial disease, diabetes mellitus, cerebrovascular accident, and osteoarthritis or rheumatoid arthritis [35, 36]. The medicines taken by the respondent were recorded from the containers by the interviewer.

### Physical performance

Physical performance was assessed at respondents’ homes and included handgrip strength (HGS), six-meter walking (6MWT), chair stand (CST), tandem balance and putting on and taking off a cardigan. HGS was measured using a grip strength dynamometer that was adjusted for hand size and recorded the grip strength to the nearest kilogram (Takei TKK 5001, Takei Scientific Instruments Co. Ltd., Tokyo, Japan). HGS was measured twice for both the left and right hand. Maximum HGS was determined by the highest grip strength. Gait speed was assessed by time in seconds doing the 6MWT which consists of walking three meters, turn around and walk three meters back as quickly as they can. Gait speed was measured in meters per second by dividing six meters by the time to do the test. The CST was used to assess the ability of individuals to rise from a chair with their arms crossed over their chest, stand up to a straight position and sit down again. This test was performed five times. The ability and the time in seconds needed to perform the CST were used for analysis. The tandem balance test is performed by placing the heel of 1 foot directly in front of the other foot, making sure that the toes of the back foot are touching the heel of the front foot. The ability to remain in tandem stand for 10 s was used for analysis. The cardigan test measured the time in seconds required to put on and take off a cardigan.

### Data analysis

Dichotomous variables were presented as number and percentage. Continuous variables were presented by mean, standard deviation if data was normally distributed or median, interquartile range (IQR) if data was skewed. Differences in population characteristics were analyzed with a chi-square test for dichotomous variables, an independent samples t-test for continuous variables and Mann-Whitney U test if continuous data was skewed. Physical performance of individuals prior to the diagnosis cancer and controls was compared using multiple logistic (CST and tandem balance test ability) and linear (HGS, gait speed, CST time, cardigan time) regression models including stepwise adjustment for possible confounders including gender and age and height, weight, current smoking and number of chronic diseases. Diagnosis of cancer was defined as the independent variable and was coded (0) no cancer and (1) cancer. Physical performance measurements were defined as the dependent variables. *P*-values of less than 0.05 were considered statistically significant. Statistical analyses were performed using Statistical Package for Social Sciences for Windows (SPSS Inc., Chicago, USA), version 22.

## Results

### Study sample

The median age of the total study sample was 68.7 [IQR 63.3–76.4] years. Table 1 shows the characteristics of the group prior to diagnosis of cancer and the control group. None of the characteristics (age, gender, smoking, alcohol, anthropometric measurements or health characteristics) differed significantly between the cancer and the control group.

**Table 1:** Characteristics of the study group prior to the diagnosis of cancer and control group

	Number	Cancer *n* = 414	Number	Controls *n* = 1321	*p*-value
Socio-demographics					
Age, years, median [IQR]	414	69.2 [63.7–75.8]	1321	68.6 [63.0–76.6]	0.498
Female	414	205 (49.5)	1321	720 (54.5)	0.076
Current smoking	369	75 (20.3)	1269	220 (16.7)	0.191
Alcohol use	369		1266		0.979
No alcohol		57 (15.4)		195 (15.4)	
Moderate/light		290 (78.6)		999 (78.9)	
(Very) excessive		22 (6.0)		72 (5.7)	
Anthropometry					
Body weight, kg, mean ± SD	362	77.9 ± 13.6	1245	78.4 ± 13.4	0.590
Height, cm, mean ± SD	360	168.7 ± 9.3	1244	168.6 ± 9.3	0.913
BMI, kg/m2, mean ± SD	359	27.4 ± 4.2	1237	27.6 ± 4.3	0.364
Health characteristics					
Nr chronic diseases, median [IQR]	414	1 [0–2]	1321	1 [0–2]	0.726
Nr of medicines, median [IQR]	332	2 [1–4]	1269	2 [0–4]	0.853
MMSE, median [IQR]	414	28 [27–29]	1321	28 [27–29]	0.064

All variables are presented in numbers (percentage) unless indicated otherwise. Alcohol was based on the Garretsen indication of present alcohol use and was categorized into three groups. *IQR* Interquartile range, *SD* Standard deviation, *MMSE* Mini Mental State Examination 0–30 points, *BMI* Body Mass Index, *kg* kilograms, *cm* centimeters, *m*^*2*^ square meters, *nr* number, *s* seconds, *m/s* meters per second

### Physical performance prior to diagnosis of cancer

Table 2 shows the mean physical performance and Table 3 shows the proportions of individuals prior to cancer diagnosis and controls on the physical performance measurements. Both tables also show the association between cancer diagnosis and physical performance in models adjusted for gender and age (model 1) and for additional potential confounders (model 2). HGS, gait speed, ability and time to perform the CST and the cardigan test did not differ between individuals prior to cancer diagnosis and controls. Individuals prior to cancer diagnosis were more likely to be able to remain in tandem position for 10 s than controls, also after adjustment for possible confounders. Results did not differ when individuals of whom the general practitioner reported cancer while the individuals themselves did not, were included in the control group.

**Table 2:** Linear regression of the association between the diagnosis of cancer and physical performance measurements

	Number	Cancer	Number	Controls	Number (regression)	Cancer, yes					
						Model 1			Model 2		
						B1	SE	*P*	B1	SE	*P*
HGS, kg	292	32.8 ± 11.8	1251	32.6 ± 11.5	1509	−0.413	0.431	0.337	−0.355	0.407	0.382
Gait speed, m/s	404	0.9 ± 0.3	1246	0.9 ± 0.3	1533	−0.007	0.015	0.640	−0.006	0.015	0.685
CST, s	376	12.1 ± 3.8	1164	12.5 ± 3.8	1434	−0.216	0.217	0.320	−0.184	0.212	0.387
Cardigan test, s	410	12.5 ± 6.9	1305	12.6 ± 6.3	1586	−0.352	0.340	0.300	−0.323	0.334	0.334

Variables are given in mean ± SD unless indicated otherwise. ‘Number regression’ is lower than the total Number of ‘Cancer’ and ‘Controls’ due to missing data in the adjusted models.  *P*-values were significant if <0.05. Model 1: adjusted for gender and age. Model 2: model 1 plus height, weight, current smoking status and number of chronic diseases. Chair stand test in seconds was only calculated for individuals who could perform the test five times. *B1* regression coefficient; *SE* standard erro, *p p*-value, *HGS* handgrip strength, *kg* kilograms, *SD* standard deviation, *m/s* meters per second, *CST* chair stand test, *s* seconds

**Table 3:** Logistic regression of the association between the diagnosis of cancer and physical performance measurements

	Number	Cancer	Number	Controls	Number (regression)	Cancer, yes					
						Model 1			Model 2		
						OR	95% CI	*p*	OR	95% CI	*p*
CST, able	404	378 (93.6)	1233	1169 (94.8)	1514	0.830	0.479–1.440	0.507	0.819	0.464–1.444	0.490
Tandem balance test, able	283	228 (80.6)	1270	983 (77.4)	1425	*1.579*	*1.069–2.334*	*0.022*	*1.573*	*1.059–2.336*	*0.025*

Variables are given in *N* (percentage) unless indicated otherwise. ‘Number regression’ is lower than the total Number of ‘Cancer’ and ‘Controls’ due to missing data in the adjusted models. *P*-values were significant if <0.05 and italicized. Model 1: adjusted for gender and age. Model 2: model 1 plus height, weight, current smoking status and number of chronic diseases. Chair stand test was scored as able if the individual was able to perform the test five times. Tandem balance test was scored as able if the individual could remain in tandem position for at least 10 s. *OR* Odds Ratio, 95% *CI* 95% Confidence Interval, *p p*-value, *CST* chair stand test

## Discussion

This longitudinal study of nationally representative older individuals showed that physical performance was not lower in individuals 2 to 4 years prior to the report of cancer compared to individuals who were not diagnosed with cancer and therefore did not support our hypothesis.

Unintentional weight loss is often one of the alarming symptoms of undiagnosed cancer. Individuals who lose weight, can lose both fat mass and muscle mass [37–41]. In this context, body weight can be a misleading marker as individuals might not lose weight if fat mass or body water increases. Low muscle mass on the other hand is an important determinant of low muscle strength, low physical performance and functional disability [42–46]. We expected that physical performance could already be lower in cancer patients prior to their diagnosis than in individuals without cancer. Lower physical performance could therewith be another alarming symptom for clinicians and individuals to pay attention to and might even be a first indicator. Possibly, there could even be a role for improving physical performance prior to cancer treatment. However, our results did not confirm our expectation. Even though a decline prior to cancer treatment has not been found, we still believe improving physical performance should be an important part of cancer treatment. Lower physical performance is associated with a higher risk on adverse outcome [13, 17–21], therefore patients could benefit from physical performance interventions prior, during and after cancer treatment, especially since a decline in physical performance is expected during the course of treatment [5, 22].

The physical performance of individuals was analyzed by use of several physical tests including HGS, gait speed, CST, time to put on and take off a cardigan and the tandem balance test. HGS, gait speed, CST and tandem balance are all commonly used tests in clinical practice as well as research and are used to determine muscle strength and physical performance [47–52]. The cardigan test is less known in clinical practice and relates to activities of daily living and relies on coordination and on the functioning of the upper extremities. Even though the tandem test is a balance test, it is influenced by core stability which is also dependent of physical function. This test might be less reproducible than other physical performance measurements [53]. The finding that individuals prior to cancer diagnosis were more likely to be able to successfully perform the tandem balance test was unexpected and cannot be explained by current literature describing insights in cancer pathophysiology.

The main strength of this study is the use of objective, well-validated measures of physical performance including handgrip strength, gait speed and balance tests. Another major strength is that this study included a large heterogeneous community-based sample of older individuals.

### Study limitations

A limitation of this study is that actual cancer diagnosis could have occurred anytime between the two measurements varying from 1 day to 4 years. Unfortunately, it was not possible to adjust for the effect of the time interval between measurement of physical performance and cancer diagnosis because the actual date of cancer diagnosis is unknown, there is only information on the date that individuals reported to have cancer during any of the measurement cycles. Furthermore, presence of cancer was based on self-report of individuals. Although there was substantial accuracy in the self-report of individuals [31, 32], self-report could still have led to under- or over reporting of cancer. Based on previous studies, underreport of cancer is most likely [32, 54] and underreport is more common in individuals without mobility limitations [32]. Thus, if there was substantial underreport of cancer, it will not have changed the results and conclusions of this study as it will have led to more individuals with higher physical performance in the cancer group. Moreover, controls were matched based on the median age of the cancer group and not per individual. However, the groups proved to be comparable in major sociodemographic characteristics. Lastly, the cancer group included all types and severity of cancer and it may be argued that some types of tumor would have a larger influence on physical performance and that more severe stages of cancer will also have a bigger impact on physical performance. This argument is supported by the study of Petrick et al. which showed that self-reported physical performance was only significantly lower in the group with lung cancer in the 3 years leading up to cancer diagnosis [22].

## Conclusions

Objectively measured physical performance of older individuals prior to cancer diagnosis was not lower than of controls who were not diagnosed with cancer. This indicates that physical performance in the time period of 2 to 4 years prior to report of cancer diagnosis is not negatively influenced by cancer, when all cancer types and stages of cancer are taken into account. Future research should focus on measurements of physical performance more closely prior to diagnosis of cancer and analyze different types and stages of cancer to be able to conclude on the trajectory of physical performance during the period before cancer is diagnosed.

## Abbreviations

6MWT: Six Meter Walking Test
BMI: Body Mass Index
CST: Chair Stand Test
HGS: Handgrip Strength
IQR: Interquartile Range
MMSE: Mini Mental State Examination
